# Supplementary material for: Imbalanced amplification: A mechanism of amplification and suppression from local imbalance of excitation and inhibition in cortical circuits
Source: PLoS Comput Biol. 2018 Mar 15;14(3):e1006048. doi: 10.1371/journal.pcbi.1006048 (PMC5871018; doi:10.1371/journal.pcbi.1006048)
Supplement: S1 Text — This supplementary text contains details of the analysis of balanced networks with connection probabilities that decay like a Gaussian with distance in physical and orientation space. (PDF) [file pcbi.1006048.s001.pdf]

Supporting Information 1 for: Imbalanced amplification: A mechanism of amplification and suppression from local imbalance of excitation and inhibition in cortical circuits

Christopher Ebsch and Robert Rosenbaum

S1 Appendix. Derivation of firing rates in balanced networks with Gaussian-shaped connectivity kernels

We now provide a more detailed analysis of firing rates in balanced networks with Gaussian-shaped connectivity kernels in physical and orientation space. This analysis mirrors that in [1].

We assume that firing rate profiles in L4 and all connectivity profiles are Gaussian-shaped in physical and orientation space. Specifically, since boundaries are periodic, they are wrapped Gaussians (see Materials and Methods). We will derive conditions on balance and conclude that, when balanced state, firing rate profiles in L2/3 are also Gaussian-shaped

Let σ_X be the width of the Gaussian-shaped firing rate profile in L4 in physical space and $\sigma_{X,\theta}$ the width in orientation space. Similarly, let α_b and $\alpha_{b,\theta}$ be the widths, in physical and orientation space, of Gaussian-shaped outgoing synaptic projections from population $b = E, I, X$. In general, we use X subscripts for L4 and E, I for L2/3 neurons.

In the Fourier domain, the firing rate profile of L4 neurons (see Materials and Methods for spatial form) is given by

$$\widetilde{r}_X(\mathbf{n}, k) = \overline{r}_X\, \widetilde{r}_{X,x}(\mathbf{n})\widetilde{r}_{X,\theta}(k)$$

where

$$\widetilde{r}_{X,x}(\mathbf{n}) = c\delta_{\mathbf{n}} + (1-c)\widetilde{G}(\mathbf{n}; \mathbf{x}_0, \sigma_X)$$

and

$$\widetilde{r}_{X,\theta}(k) = c_\theta \delta_k + (1-c_\theta)\widetilde{g}(k; \theta_0, \sigma_{X,\theta})$$

where $\overline{r}_X = \widetilde{r}_X(\mathbf{0}, 0)$ is the average rate,

$$\widetilde{g}(k; m, \sigma) = \exp\left(-2\pi^2\sigma^2 k^2 - 2\pi k m i\right)$$

is the kth Fourier coefficient of a one-dimensional wrapped Gaussian centered at m with width parameter σ,

$$\widetilde{G}(\mathbf{n}; \mathbf{m}, \sigma) = \exp\left(-2\pi^2\sigma^2\|\mathbf{n}\|^2 - 2\pi i \mathbf{n}\cdot\mathbf{m}\right)$$

is the same, but in two dimensions, δ_k is the Kronecker delta function (equal to 1 when $k = 0$ and 0 otherwise), and $\delta_{\mathbf{n}}$ is the same in two dimensions (equal to 1 with $\mathbf{n} = (0, 0)$). Similarly, connectivity in the Fourier domain is quantified by the matrices, $\widetilde{W}(\mathbf{n}, k)$ and $W_X(\mathbf{n}, k)$ whose structure is given in the main text and whose entries are given by

$$\widetilde{w}_{ab}(\mathbf{n}, k) = \overline{w}_{ab} G(\mathbf{n}; \mathbf{0}, \alpha_b) g(k; 0, \alpha_{b,\theta})$$

where $\overline{w}_{ab} = \widetilde{w}_{ab}(\mathbf{0}, 0) = \epsilon J_{ab} p_{ab} N_b$ is the average connection strength (scaled by ϵ as usual).

As indicated in the main text, the convolutions that determine the shape of external input, $\mathbf{X}(\mathbf{x}, \theta)$, become multiplication in the Fourier domain so that

$$\widetilde{X}(\mathbf{n}, k) = \widetilde{W}_X(\mathbf{n}, k)\widetilde{r}_X(\mathbf{n}, k) = \overline{\mathbf{X}}\,\widetilde{\mathbf{X}}_x(\mathbf{n})\widetilde{\mathbf{X}}_\theta(k)$$

where $\overline{\mathbf{X}} = \overline{W}_X \overline{r}_X = [\overline{X}_E \;\; \overline{X}_I]^T$ is the mean external input,

$$\widetilde{\mathbf{X}}_x(\mathbf{n}) = c\delta_{\mathbf{n}} + (1-c)G(\mathbf{n}; \mathbf{x}_0, \beta_X)$$

is the physical-space component, and

$$\widetilde{\mathbf{X}}_\theta(k) = c_\theta \delta_k + (1-c_\theta) g(k; \theta_0, \beta_{X,\theta})$$

is the orientation-space component. Here, $\beta_X^2 = \sigma_X^2 + \alpha_X^2$ is the width of feedforward input in physical space, $\beta_{X,\theta}^2 = \sigma_{X,\theta}^2 + \alpha_{X,\theta}^2$ in orientation space, and $\overline{X}_a = \overline{w}_{aX}\overline{r}_X$ is the mean external input to population $a = E, I$. Hence, the external input is Gaussian shaped with widths β_X and $\beta_{X,\theta}$ in physical and orientation space.

Inverting $\widetilde{W}(\mathbf{n}, k)$ and plugging it into Eq. (13) of the main text along with the expression for $\widetilde{X}(\mathbf{n}, k)$ above, we find that $\widetilde{\mathbf{r}}(\mathbf{n}, k) = [\widetilde{r}_E(\mathbf{n}, k) \;\; r_I(\mathbf{n}, k)]^T$ with

$$\widetilde{r}_a(\mathbf{n}, k) = \overline{r}_E\, \widetilde{r}_{a,x}(\mathbf{n}, k) r_{a,\theta}(\mathbf{n}, k) \tag{S.1}$$

for $a = E, I$ where $\overline{\mathbf{r}} = [\overline{r}_E \;\; \overline{r}_I] = -\overline{W}^{-1}\overline{\mathbf{X}}$ are the mean firing rates,

$$\widetilde{r}_{a,x}(\mathbf{n}) = c\delta_{\mathbf{n}} + (1-c)G(\mathbf{n}; \mathbf{x}_0, \sigma_a^2)$$

is the physical-space component, and

$$\widetilde{r}_{a,\theta}(k) = c_\theta \delta_k + (1-c_\theta) g(k; \theta_0, \sigma_{X,\theta})$$

is the orientation-space component. Here,

$$\sigma_a^2 = \beta_X^2 - \alpha_a^2$$

and

$$\sigma_{a,\theta}^2 = \beta_X^2 - \alpha_{a,\theta}^2$$

for $a = E, I$. Also, $\overline{W} = \widetilde{W}(\mathbf{0}, 0)$ and $\overline{\mathbf{X}} = \widetilde{\mathbf{X}}(\mathbf{0}, 0)$ are the average recurrent connection strengths and external inputs. This completes the derivation of Eqs. (15).

Whenever $\sigma_a^2 = \beta_X^2 - \alpha_a^2 > 0$ and $\sigma_{a,\theta}^2 = \beta_X^2 - \alpha_{a,\theta}^2 > 0$, Eq. (S.1) gives a well-defined Fourier series that produced Gaussian-shaped firing rate profiles, $\mathbf{r}(\mathbf{x}, \theta)$, with widths given by σ_a and $\sigma_{a,\theta}$. Hence, when external input is broader than recurrent connections ($\beta_X > \alpha_a$, etc.), balanced network theory predicts Gaussian-shaped firing rate profiles in the recurrent network.

When external input is narrower than recurrent connections, $\sigma_a \leq 0$ or $\sigma_{a,\theta} \leq 0$ for $a = E$ and/or $a = I$, the Fourier series with coefficients given in Eq. (S.1) does not converge since the coefficients diverge to ∞ as $\|n\|$ or k go to ∞. This indicates the lack of a solution to the integral equation, Eq. (11), from the main text [2] and therefore a break in balance [1] whenever $\beta_X \leq \alpha_a$ and/or $\beta_{X,\theta} \leq \alpha_{a,\theta}$ for $a = E$ and/or $a = I$.

References

[1] R Rosenbaum and B Doiron. Balanced networks of spiking neurons with spatially dependent recurrent connections. *Phys Rev X*, 4(2):021039, 2014.

[2] F G Tricomi. *Integral equations.* Interscience, New York, 1957.
