# Supplementary material for: Imbalanced amplification: A mechanism of amplification and suppression from local imbalance of excitation and inhibition in cortical circuits
Source: PLoS Comput Biol. 2018 Mar 15;14(3):e1006048. doi: 10.1371/journal.pcbi.1006048 (PMC5871018; doi:10.1371/journal.pcbi.1006048)
Supplement: S2 Text — This supplementary text contains simulations and analysis of balance and imbalanced amplification in a network model with conductance-based synapses. (PDF) [file pcbi.1006048.s002.pdf]

Supporting Information 2 for: Imbalanced amplification: A mechanism of amplification and suppression from local imbalance of excitation and inhibition in cortical circuits

Christopher Ebsch and Robert Rosenbaum

S2 Appendix. Balance and imbalanced amplification in a model with conductance-based synaptic and optogenetic inputs

In the main text, we considered a neuron model with current-based synapses and a current-based model of optogenetic stimulation. We now show that our results are easily adapted to a conductance-based adaptive exponential integrate-and-fire (AdEx) model. This model is identical to the one used in the main text, but the voltage-independent input current, $I_j^a(t)$ to neuron $j = 1, \ldots, N_a$ in population $a = E, I$ from that model is replaced by a voltage-dependent input current given by

$$I_j^a(V,t) = g_j^{aE}(t)[E_E - V(t)] + g_j^{aI}(t)[E_I - V(t)] + g_j^{aX}(t)[E_E - V(t)] + g_j^{aChR2}(t)[E_{ChR2} - V(t)]$$

where $g_j^{ab}(t)$ is the conductance of type $b = E, I, X, ChR2$ and E_b is the associated reversal potential. Note that $g_j^{IChR2}(t) = 0$ since inhibitory neurons do note express ChR2 in our model. The synaptic conductances ($b = E, I, X$) are given by

$$g_j^{ab}(t) = \sum_{k=1}^{N_b} J_{jk}^{ab} \sum_n \eta_b(t - t_n^{b,k}) \tag{S.1}$$

where $a = E, I$ is the postsynaptic cell population, $b = E, I, X$ is the presynaptic cell population, and $\eta_b(t) = (1/\tau_b)e^{-t/\tau_b}\Theta(t)$ is a postsynaptic conductance waveform (identical to the current waveforms used in the main text). When optogenetic stimulation is on, $g_j^{EChR2}(t) = s$ for expressing neurons, j, but $g_j^{IChR2}(t) = 0$ to represent pyramidal-cell specific expression of ChR2.

The mean-field analysis in the main text relies on an assumption that currents add linearly, which is not true for this conductance-based model. However, the analysis can be recovered under a formalism in which the conductance-based model is approximated by a current-based one [1, 2, 3]. We review this approximation further below, but first give the conclusions and resulting firing rate approximations.

For the two-population case (one excitatory and one inhibitory population, as in Fig. 1 and the surrounding discussion, this approximation gives rise to an approximate "effective" current,

$$\mathbf{I}^{eff} = \frac{1}{\epsilon}\left[W^{eff}\mathbf{r} + \mathbf{X}^{eff}\right], \tag{S.2}$$

which generalizes Eq. (1) from the main text. Here,

$$\epsilon = \frac{1}{(E_E - V_0)K_{EX}J_{EX}}$$

$$\mathbf{X} = W_X^{eff} r_X + \left[\begin{array}{c} s^{eff} \\ 0 \end{array} \right],$$

$$W^{eff} = \left[\begin{array}{cc} w_{EE}^{eff} & w_{EI}^{eff} \\ w_{IE}^{eff} & w_{II}^{eff} \end{array} \right] \text{ and } W_X = \left[\begin{array}{c} w_{EX}^{eff} \\ w_{IX}^{eff} \end{array} \right],$$

$$w_{ab}^{eff} = \frac{(E_b - V_0)K_{ab}J_{ab}}{(E_E - V_0)K_{EX}J_{EX}},$$

and

$$s^{eff} = (E_{ChR2} - V_0)s.$$

This is identical to the current-based analysis, except connection strengths and optogenetic currents are scaled by the distance of their associated reversal potentials to V_0. An identical generalization applies to the analysis in which excitatory neurons are broken into ChR2-expressing and non-expressing populations (as in Fig. 2 and surrounding discussion) and to spatially extended networks (as in later figures). Here, V_0 represents the mean membrane potential across neurons and time, which we computed from simulations. Hence, this analysis requires that simulations be performed first, which reduces its applicability for predictive purposes, but still works for understanding how balance and imbalanced amplification occur in conductance-based models, which is our main goal here.

The analysis of firing rates then proceeds as before. Balanced firing rates are given by

$$\mathbf{r} = -[W^{eff}]^{-1}\mathbf{X}^{eff}$$

which only exist when W^{eff} is invertible (or when W is not invertible, but $\mathbf{X}$ is in its column space). The linear correction to balance is given by

$$\mathbf{r} = [\epsilon D - W^{eff}]^{-1}\mathbf{X}^{eff}$$

where D is the same as before. We applied this analysis to conductance-based versions of Figs. 1-2 from the main text and found that it was similarly accurate and imbalanced amplification was similarly observed (Supporting Figure 1).

We now review the approximation from which the effective current in Eq. (S.2) was derived. Consider the AdEx membrane potential dynamics defined by

$$C_m \frac{dV}{dt} = -g_L(V - E_L) + I(V,t) + g_L \Delta_T \exp[(V - V_T)/\Delta_T] - w$$

where $I(V,t)$ is the conductance-based input defined above. This can be re-written as

$$C_m \frac{dV}{dt} = -g_{eff}(t)(V - V_0) + I_{eff}(t) + g_L \Delta_T \exp[(V - V_T)/\Delta_T] - w$$

where

$$g_{eff}(t) = g_L + g_E(t) + g_I(t) + g_X(t)$$

and

$$I_{eff}(t) = g_L[E_L - V_0] + g_E(t)[E_E - V_0] + g_I(t)[E_I - V_0] + g_X(t)[E_E - V_0] + g_{ChR2}(t)[E_{ChR2} - V_0] \quad \text{(S.3)}$$

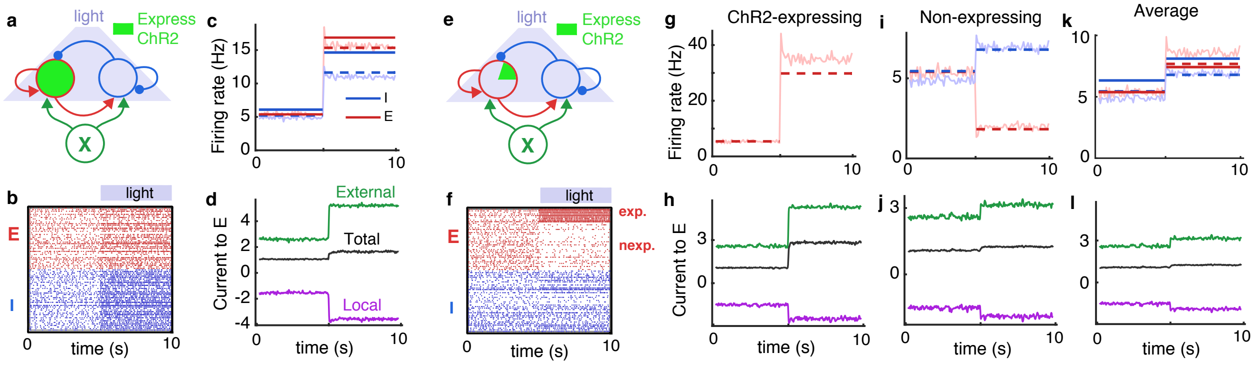

Supporting Figure 1: **Figures 1 and 2 reproduced in a model with conductance based synapses. a-d)** Same as Fig. 1 a-d of the main text, but with a conductance-based synapse model. **g-l)** Same as Fig. 2a-h of the main text, but with a conductance-based model. Reversal potentials were $E_E = E_{ChR2} =$ 0mV and $E_I = -100$mV. Connections were chosen randomly in the same way as in the main text with connection weights chosen to give similar postsynaptic potential amplitudes when the membrane potential starts near -65mV. This was achieved at $J_{EE} = 0.0062C_m$, $J_{EI} = J_{II} = 0.1675C_m$, $J_{IE} = 0.0129C_m$, $J_{EX} = J_{IX} = 0.0072C_m$. The optogenetic conductance was also chosen to produce a similar current at -65mV by setting $s = 0.0308C_m/ms$.

where V_0 can be any arbitrary reference voltage. Note that $I_{eff}(t)$ is not voltage-dependent, but the model is still conductance-based due to the appearance of the $g_{eff}(t)(V - V_0)$ term. The idea behind the approximation is as follows: When V_0 is chosen close to the steady-state mean membrane potential, the balance of excitatory and inhibitory currents implies that $I_{eff}(t)$ is much smaller on average than the positive excitatory and negative inhibitory contributions to it. Thus, the variability in the conductances, $g_b(t)$, contribute much to $I_{eff}(t)$. On the other hand, the excitatory and inhibitory contributions to $g_{eff}(t)$ are both large ($\mathcal{O}(1/\epsilon)$) and positive. Thus their mean values are much larger than their variability, even in the balanced regime. This argument motivates the substitution of $g_{eff}(t)$ with its mean value, leading to current-based approximation

$$C_m \frac{dV}{dt} = -\overline{g_{eff}}(V - V_0) + I_{eff}(t)$$

where

$$\overline{g_{eff}} = g_L + \overline{g_E} + \overline{g_I} + \overline{g_X} + \overline{g_{ChR2}}$$

is the steady-state mean conductance. Taking means in Eqs. (S.3) and Eq. (S.1) gives Eq. (S.2). Note that $\overline{g_{eff}}$ is $\mathcal{O}(1/\epsilon)$, so neurons become highly conductive (leaky) for small ϵ [1, 2, 3]. As a consequence, membrane potentials track synaptic nearly perfectly (without substantial lag or filtering) when ϵ is small because the effective membrane time constant of neurons is $\tau_{eff} = C_m/g_{eff} \sim \mathcal{O}(\epsilon)$. However, the timescale of network dynamics is still limited by the synaptic time constants, τ_b, which determine the timescales of the synaptic conductance waveforms, $\eta_b(t)$.

References

[1] A Destexhe and D Paré. Impact of network activity on the integrative properties of neocortical pyramidal neurons in vivo. *J Neurophysiol*, 81(4):1531–47, 1999.

[2] A Destexhe, M Rudolph, and D Paré. The high-conductance state of neocortical neurons in vivo. *Nat Rev Neurosci*, 4(4):739–51, 2003.

[3] A Kuhn, A Aertsen, and S Rotter. Neuronal integration of synaptic input in the fluctuation-driven regime. *J Neurosci*, 24(10):2345–56, 2004.
